# Reluctant educators and self‐advocates: Older trans adults’ experiences of health‐care services and practitioners in seeking gender‐affirming services

**DOI:** 10.1111/hex.13104

**Published:** 2020-07-16

**Authors:** Paul Willis, Christine Dobbs, Elizabeth Evans, Michele Raithby, Jenny‐Anne Bishop

**Affiliations:** ^1^ School for Policy Studies University of Bristol Bristol UK; ^2^ Centre for Innovative Ageing College of Human and Health Sciences Swansea University Swansea UK; ^3^ Unique Transgender Network North Wales Wales UK

**Keywords:** ageing, equality, gender, gender identity, general practitioners, health care, older age, trans, transgender, trans‐related health care

## Abstract

**Background:**

Trans‐identifying individuals experience unique barriers and challenges in negotiating health‐care systems due to the cisnormative attitudes and practices which obstruct the receipt of trans‐inclusive care. To date, there has been little exploration of older trans consumers’ experiences of contemporary health‐care services when seeking to transition medically in later life.

**Objectives:**

Qualitative findings are presented from a study of trans ageing and trans‐related health and social care needs in Wales, UK (2016‐18). The objectives are to (1) examine supportive and obstructive points of interaction with health‐care professionals, and (2) identify key learning messages for improving trans‐related health care from the perspectives of trans‐identifying adults in later life.

**Design:**

Trans‐identifying participants self‐selected to take part in two interviews—a life‐history interview and a semi‐structured interview. Interview data were analysed thematically using the framework method approach.

**Setting and participants:**

This paper focuses on the accounts of 19 participants (50‐74 years of age) who identified as trans and were seeking to transition medically in mid‐ to later life.

**Results:**

Findings indicate how older trans patients are positioned as reluctant educators for GPs in primary care settings and illustrate the transphobic practices and cisnormative assumptions encountered across health‐care interactions and systems that impede their journey of transitioning in later life.

**Discussion and conclusions:**

Messages from this study speak to the importance of improving professionals’ knowledge of gender identity diversity across the life course and making changes at a systemic level in redressing cisnormative assumptions and systems that reinforce inequities on the basis of gender identity.

## 1 INTRODUCTION

The views and experiences of health‐care consumers are paramount to improving the quality and provision of health‐care services, with growing awareness of the importance of co‐creation with consumer groups in delivering good services.^1^, ^2^, ^3^ This paper focuses on a consumer group whose unique life accounts have received little recognition in health‐care research—the experiences of older trans‐identifying^1^ adults accessing trans‐related health‐care services. We present qualitative findings from a study of trans ageing and care needs in Wales (2016‐18) and examine trans adults’ (50‐74 years of age) accounts of their interactions with health‐care professionals as part of their journey of gender transitioning. ‘Transitioning’ refers to the processes trans individuals undertake in changing their presentation and expression to align with their gender identity. This may involve social transitions (eg dressing and self‐presentation) and/or medical transitions through accessing gender‐affirming treatments (eg prescribed hormones or surgical treatments).^4^ For many, this is a life‐long journey. In the United Kingdom (UK), a 2016 Parliamentary Committee noted that the National Health Service (NHS) was letting down trans individuals, and there was considerable evidence of discriminatory practice ‘in breach of the Equality Act’ (p. 35).^5^ In England and Wales, the Equality Act 2010 promotes non‐discriminatory services, inclusive of health‐care services. Trans‐identifying individuals are protected under the characteristic of ‘gender reassignment’ (s. 7(1)).

UK and European survey findings indicate hostile climates towards trans citizens as they experience victimization, hate crime and hostility because of their gender identity.^6^, ^7^, ^8^ Living in a hostile environment impacts on the confidence of trans citizens in being visible and ‘out’ to others. From the 2018 UK Government survey of 100 000 + LGBT respondents, more than half of trans respondents (59% trans women; 56% trans men) reported they avoided expressing their gender identity.^9^ Epidemiological data show that as a minority group, they are substantially affected by adverse health outcomes, including mental health and substance misuse.^10^ For adults 50 + years of age, a US survey of LGB (and trans) adults found that trans respondents were at significantly higher risk of poor physical health, disability, depressive symptomatology and perceived stress compared with cisgender peers (ie individuals whose gender identity configures with the sex assigned to them at birth).^11^

Health‐care services are not immune from the hostile environments in which they are located. Reported structural and interpersonal barriers in health‐care settings include being placed in inappropriate hospital wards and not having access to suitable bathrooms,^12^ encountering abusive treatment from medical staff, improper use of gender pronouns, binary gender language recorded on documents^13^, ^14^ and patients not receiving the care required as they do not fit into a binary gender model, that is male/female.^15^ Anti‐trans attitudes can lead to more severe outcomes such as the denial of trans‐specific treatments.^16^

Negative experiences of services in earlier life will impact detrimentally on current perceptions of providers and diminish older trans patients’ trust and confidence.^15^ In the UK, over a third (38%) of trans‐identifying respondents in the National LGBT Survey reported negative experiences of general health‐care services.^9^ Amongst respondents accessing gender identity clinics (GICs), 80% reported accessing had not been easy with 68% indicating waiting lists were too long. Other UK surveys of trans respondents tell a more nuanced story with reports of insulting language from health‐care professionals, including general practitioners (GPs), and GPs being unwilling to support with trans‐related health care.^12^, ^17^, ^18^ Anecdotal accounts suggest recurring problems with GPs as gatekeepers to GICs and a wider lack of knowledge about referral pathways and treatments^5^ alongside reluctance from GPs and practice managers to take part in trans‐related research.^5^ There is a gap in current research conveying clinicians’, including GPs’, views on current treatment pathways and options for older adults.

For trans individuals seeking to transition medically, GICs are tertiary services that provide access to gender‐affirming treatments and surgeries in the UK.^4^ Access to these services hinges on the receipt of a diagnosis of ‘gender dysphoria’, as listed in the Diagnostic and Statistical Manual of Mental Disorders (DSM‐5).^16^, ^19^ To obtain treatments, trans individuals are required to conform to medical expectations of appropriate binary gendered behaviour and presentation; to present in a gender‐ambiguous way risks denial of treatment.^20^ Within biomedical discourse, this diagnostic label positions ‘trans’ as a condition to be ‘fixed or resolved’ (p. 9).^16^ More recently, the World Health Organization has reclassified the term ‘dysphoria’ as ‘gender incongruence’ and removed this diagnosis from being listed as a mental disorder.^21^ It remains to be seen whether this attempt to de‐pathologize trans identities trickles down to inform clinicians’ understanding.

The knowledge gaps of gatekeepers and long waiting lists are two major obstacles to accessing GICs. Waiting times for GICs are reportedly the longest of any specialist service in the UK.^4^ A UK study of 74 trans individuals (50 + years) accessing a GIC over a 30‐month period found that the majority of service users were trans women, 50% had sourced hormone treatments via the Internet, and 28% had obtained this treatment without medical advice.^22^ At the same time, patients taking cross‐sex hormones were significantly less anxious and reported higher levels of self‐esteem than those who did not. This supports other tentative conclusions that the receipt of hormone treatment can reduce some indicators of psycho‐social distress and anxiety disorders.^23^, ^24^ In this paper, our objectives are (1) to examine supportive and obstructive points of interaction with health‐care professionals, and (2) to identify key learning messages for improving trans‐related health care from the perspectives of trans‐identifying adults in later life. Our research question is, ‘What are the experiences of trans‐identifying older adults in accessing and receiving health‐care services on their journey of transitioning medically?’

## 2 KEY CONCEPTS

There are several key concepts underpinning this study. The term ‘transphobia’ signifies expressions and attitudes of aversion and hostility towards trans individuals and people who are gender non‐conforming,^25^ manifesting in the form of microaggressions, such as the intentional misgendering of another person, to overtly hostile acts such as physical violence.^4^ Transphobic beliefs are underpinned by cisgenderist ideology. Cisgenderism is the dominant cultural belief that individuals who self‐define their gender are perceived to be socially inferior or lacking validity compared to those whose sex assigned at birth matches their gender identity.^26^, ^27^ The distinction drawn between cisgender people (or non‐transgender) and trans individuals is a form of cisgenderism that excludes people who identify with more than one gender or identify outside the binary divide between male and female.^27^ Everyday examples of cisgenderism at a service level include refusal to recognize a patient's gender identity or, at an institutional level, imposing medical or legal requirements that trans citizens must meet to be recognized fully.

To counterbalance the problem‐saturated health narrative attached to trans identities, Reisner *et al* ^10^ champion a gender affirmation model of public health. This model validates an individual's gender identity across four dimensions: social (eg recognition by name); psychological (eg self‐perceptions and self‐identity); medical (eg access to treatments); and legal (eg procedures for changing names). These authors acknowledge that this model has less applicability to people who identify outside the male/female binary. However, it does provide a foundation for reforming service delivery towards a more trans‐inclusive approach.^10^ From a gender affirmative perspective, older trans adults who are dependent on others for care and support may need assistance with a range of everyday decisions and actions, including how to express their gender identity in person and on formal documentation, adhering to hormone prescriptions and how to access treatments, inclusive of post‐surgical procedures and daily care.^27^

## 3 METHODS

Twenty‐two (22) trans‐identifying individuals participated in two interviews each, leading to a total of 43 interviews (one person participated in one interview only). A purposive sampling approach was adopted for interview recruitment. Advertisements for participation were circulated through trans community groups, online forums and social networks, identified through the project's Critical Reference Group. The project team also participated in local trans and LGBT pride‐related events over a 12‐month period to advertise the study. Participants were self‐selecting. Criteria for participation were as follows: (i) identifying as trans or gender non‐conforming; (ii) residing in Wales; and (iii) 50 + years of age. Two men resided in England at the time of interview; they were included as the majority of interviews were with women (15) and the accounts of men who had transitioned were missing. The minimum age of participation reflected the policy approach in Wales in which citizens are deemed to be eligible for accessing some services for older adults from 50 + years.

Ethical approval was obtained through the Host University—the College of Human and Health Sciences Research Ethics Committee. Potential interviewees received an information sheet, and informed consent was obtained prior to the first interview. Recalling experiences of living in cisnormative environments can evoke a range of emotional responses, including distress, and ways of supporting participants were carefully considered. Participants were debriefed after each interview and received a follow‐up phone call from the research officer between the two interviews. At the end of the first interview, participants were provided with a debrief sheet listing local support services and helplines for additional support. If needed, participants could be referred to a BACP‐registered counsellor (who identified as trans) for additional support. To enhance participant autonomy, participants were sent electronic copies of their transcripts and given a set time period to review and edit details if they wished prior to analysis.

In this paper, we concentrate on the accounts of 19 participants who were transitioning through medical means—the other three participants identified as genderqueer or crossdressers and were not seeking to transition. We selected these 19 participants because of their first‐hand experience of accessing trans‐related health care, including local GPs, mental health professionals and GICs. The first interview was unstructured and followed a life‐history format. During these interviews, participants shared their life story, which included key turning points of seeking professional support and making first contact with primary services, including GPs. The second interview was semi‐structured and included questions on topics identified in the literature including social network membership; experiences (recent and past) and expectations of health and social care services and professionals; and three wishes for change. Participants expanded on their current or most recent experiences of accessing trans‐related health care and associated issues and concerns. Interviews ran between 1.5 and 3 hours each, and most occurred in participants’ homes, at their invitation.

Interviews were audio‐recorded, transcribed and analysed using the framework approach. This approach is one method of qualitative data management and analysis applied in social policy and health‐care research.^28^ We followed the step‐by‐step guidance produced by NatCEN.^29^ The first stage is building a thematic framework: four team members conducted an independent coding exercise on six participants’ transcripts to identify manifest themes. Team members met to share and compare initial codes and agree the thematic categories and sub‐categories to be applied to all data. The thirteen categories are listed in Table 1. The agreed framework was created in NVivo (qualitative software program), and participants’ transcripts were assigned a case number and key attributes. The next stage was coding data: transcripts were coded by two team members using the framework categories. This was followed by summarizing data within NVivo: codes (or ‘nodes’ in NVivo) were charted across the framework by summarizing the relevant, coded data per category and indicating illustrative quotes. The final stage was interpreting the data: we applied a thematic method by which categories were selectively coded with two team members checking that summaries adhered to charted data. From the summaries, core themes were generated that conveyed collective, more explanatory accounts of participants’ individual experiences.

**TABLE 1:** Categories and sub‐categories created for the framework of data analysis

Categories	Sub‐categories
1. About me	1.1 Social and familial background
	1.2 Pets
	1.3 Trans self‐definitions
	1.4 Current relationships—partner
	1.5 Current relationships—family
	1.6 Current relationships—friends
	1.5 Who provides support in current life
	1.6 Other
2. Becoming me	2.1. Barriers in later life
	2.2. Facilitators in later life
	2.3 Opposite‐gender relationships
	2.4 Social pressures
	2.5 Accessing hormones
	2.6 Life philosophy and beliefs
	2.7 No social reference points (historic)
	2.8 Other
3. Coming out as trans	3.1 Attitudes and reactions from family members
	3.2 Attitudes and reactions from non‐family members
	3.3 Hiding trans self from others
	3.4 Too risky to be out
	3.5 Other
4. Care experiences, concerns and expectations	4.1 Care—dementia
	4.2 Care—expectations in later life
	4.3 GIC—positive experiences
	4.4 GIC—negative experiences
	4.5 Experiences with GP—negative
	4.6 Experiences with GP—positive
	4.7 Hospital staff—negative experiences
	4.8 Seeking medical care—extreme measures
	4.9 Medical care—historic
	4.10 Mental health staff—experiences of
	4.11 Health‐care system—negative experiences
	4.12 Other
5. Dressing and presenting as me	5.1 Dressing as ‘me’
	5.2 Dressing in secrecy
	5.3 Perceptions of other trans individuals
	5.4 Other
6. First encounters	6.1 First encounter with the idea of trans
	6.2 First encounters with trans groups/communities
	6.3 Other
7. Growing older	7.1 Growing older—barriers
	7.2 Growing older—discussion with significant others
	7.3 Growing older—financial
	7.4 Growing older—health
	7.5 Responses to David Bowie quotation: ‘ageing as an extraordinary experience where you become the person you always should have been’
	7.6 Comparisons—younger versus older
	7.7 Regrets in later life
	7.8 Other
8. Mental health and well‐being	8.1 Mental health isolation and/or depression
	8.2 Mental health self‐harm
	8.3 Mental health‐suicidal thoughts and actions
	8.4 Internal struggle and external battle
	8.5 Feeling shame in childhood and adolescence
	8.6 Feeling shame in adulthood
	8.7 Other
9. Challenges and barriers: childhood and adolescence	9.1 Expectations attached to gender assigned at birth
	9.2 Physical health poor
	9.3 Puberty—impact
	9.4 Abuse and neglect in childhood and/or adolescence
	9.5 Other challenging experiences
	9.6 Other
10. Sexuality	10.1 Sexuality—adolescence
	10.2 Sexuality—adulthood
	10.3 Intersection of sexual/gender identity
	10.4 Other
11. Social media	11. Social media usage—purpose and platforms
12. Hate crime, discrimination, transphobia	12.1 Being outed
	12.2 Misgendering
	12.3 Discrimination and harassment at work
	12.4 Discrimination and harassment—other experiences
	12.5 Hate crime
	12.6 Identifying safe spaces
	12.7 Other
13. Three wishes question	What three things would you wish to change in current health and social care services for older trans people?

Table 2 summarizes participants’ key characteristics. The majority were reliant on public‐funded health care (free through the NHS). Four participants had privately funded gender‐affirming treatments but still needed to access local public services. Below, we focus on experiences of health‐care services within the last 10 years for currency. Extracts are presented using participant‐selected pseudonyms.

**TABLE 2:** Key characteristics of interview participants

N = 22	
Age range	50‐74 years 50‐59 years (10) 60‐69 years (11) 70 + years (1)
Ethnic background	White—born in the UK (20) White—born outside the UK (2)
Current residence^a^	Rural area (town, village or hamlet/ isolated dwelling) (10) Urban area (city or suburb) (12)
Transitioning (socially and/or medically)	Trans women (15) Trans men (4) Not seeking to transition/non‐gender conforming (3) (eg identifying as a crossdresser)—not included in the below findings.

^a^ Two participants lived in England at the time of interviews.

## 4 RESULTS

We report three core themes. Some of the findings reflect identified problems with the current system of referral for trans patients in Wales. Through its devolved powers, the Welsh Government administers its own public health‐care services, which have resulted in different pathways for accessing GICs compared to pathways in England or Scotland. At the time of the study, the Welsh protocol involved referring all Welsh‐residing patients to a GIC in England via a mental health assessor in Wales acting as a local gatekeeper. This gatekeeper determined whether individuals were ‘gender dysphoric’. During the course of this study, the established pathway to accessing GICs has been under review, with a new gender identity service recently launched to provide trans‐related health care closer to home for citizens living in Wales.^30^ Under the new service, launched September 2019, patients are now directly referred by their GP or another appropriate health professional to the Welsh Gender Service Clinic.

### 4.1 General practitioners as inconsistent allies

The most prominent theme was the wide variation in knowledge levels amongst GPs about trans people's needs, available treatments and referral pathways to the GIC. Unsurprisingly, increasing trans‐related knowledge of GPs was the most frequently expressed ‘wish’ when identifying recommendations for change to services.

#### 4.1.1 Trans patients as reluctant educators

Gaps in GPs’ knowledge generated frustration for participants who often felt that the onus was on them to find out information to inform their GP about what should be done or to push for treatments or referrals to the GIC. Participants were reluctant educators for their GPs, for example bringing information to their GP about which hormones to prescribe or clinical services to refer to for assessments. Reflecting on their first conversations with GPs about their gender identity, several participants described mixed responses. Some GPs appeared like ‘rabbits in headlights’, while others expressed uncertainty on how to proceed. Enduring an extended wait for follow‐up was a common theme:

First time I ever went in there [GP clinic], I just said I’d got these overwhelming feelings I want to be a woman … I’ve had it all my life … I’m fifty‐odd now and it’s not going away … I really feel now I’ve got to go for it. And the first thing she said is, ‘What do I do about that, then?’ ‘I don’t know,’ I said […] I’d seen all this stuff about [clinic in England], so I said to her, ‘I don’t know what to say, really. You’re the expert”, kind of thing. ‘I do know there’s this clinic in [….]. Is there any chance at being referred to them?’ And she was all a bit, ‘Oh well, I suppose I can write to them. I don’t know’. I never heard anything for about two or three months. (Sophie, 58)

Several participants who had decided to commence transitioning medically in their 40s and 50s were worried about getting older and ‘losing time’ on their journey as they did not have ‘years and years to wait’. For the majority of participants who were transitioning, this decision had been made over a long period of time, sometimes several decades, adding to their sense of anticipation. Participants perceived GPs as failing to educate themselves about trans patients’ needs in the way they would for other health problems, such as cancer. The lack of knowledge about gender‐affirming treatments and GICs can manifest as an unwillingness to investigate further and generate further delays.

Knowledge gaps persisted for participants who were seeking continuing health care post‐surgery. Sophia (a different participant to Sophie) had received private surgical treatment overseas in 2004 but needed to access her local GP for advice on switching prescribed hormones:

The doctor said to me, ‘Well, I may look into it for you, but I can’t promise anything’, and I said, ‘Well, okay, do you mind if I look into it, and then I come back to you with a name or a suggestion’, or, and he said, ‘Okay, yes. I’m open to that’. (Sophia, 53)

Prescribing hormone treatment and monitoring hormone levels were seen as being relatively straightforward care for GPs to provide. However, it was thought that some GPs did not know how to do this and were anxious about prescribing, preferring to rely on endocrinologists, of whom there are few in Wales. Moreover, GPs were sometimes ignorant about care and funding entitlements available through the NHS for treatments and surgery. The onus was on trans patients to educate and to push for treatments:

I started to masculinise quite quickly. So I thought well, okay, I better go and try and get my surgery now. So I went to the doctor and I said, ‘Do you know how I go about funding?’ And they said, ‘don’t know’. That was it … and there was a silence after, and I thought they’re going to say now, ‘But I’ll find out for you’, or, ‘I know of somebody who might know’, but no, nothing. They didn’t want to know. (Richard, 63)

Asking ‘pertinent questions’ based on information obtained through the Internet was one strategy for putting pressure on GPs to acquire more information. In this context, the trans individual's superior knowledge signifies a subtle shift in power from the doctor to the patient, which sits more comfortably with some GPs than others.

#### 4.1.2 Supportive and affirming responses

Some GPs were described as ‘highly supportive’ in the level of care provided. Three participants shared affirmative responses from their GPs when presenting as themselves:

My GP, she’s been really supportive, and has genuinely you know, been there for me … The first time I went as Barbara she looked at me and went, ‘Amazing…,Where is that person who sat there is his, in his Barbour jacket, with his, sort of chin in his boots?’ (Barbara, 69)

One participant, James, had developed deep mistrust for health‐care professionals based on a history of health complications arising from genital surgeries received in the 1970s and 1990s. However, a more recent GP experience had regained his trust and confidence through her consistent care and support for him. For others, a lack of knowledge about trans‐related health care was not always a barrier to receiving good care:

[GP had not] heard of anyone transgender before, … but again she found out a lot of things for me. I told her that… I needed to transition and explained my situation to her… She was very supportive from the word go. (Gabriella, 56)

Others described examples of GPs being supportive through ensuring access to funding for treatments, being perceived to be doing more than they needed to and delivering on promised information and referrals.

### 4.2 Discriminatory and negative responses from health‐care professionals and services

A small group of participants relayed experiences of discriminatory responses from health‐care professionals, including GPs, district nurses and general practice staff. After an initial consultation, Claire's GP had insisted that she pay for her own hormone prescriptions (prescriptions are free in Wales):

She [GP] said to me, ‘Well, I don’t agree with the NHS paying for medication of this sort for people like you’. I was really taken aback by that… she totally cut my legs from under me. With a very meek voice, I said, ‘I’m willing to pay for them’. ‘Right,’ she says, ‘If you’re willing to pay for them’ (Claire, 67)

This encounter reveals a GP’s decision making on personal, moral grounds, rather than professional, ethically informed grounds. Several participants had encountered hostile responses when accessing primary care services:

After the operation [genital reconstruction surgery] I had to go back to my surgery and that’s when the nurse in the surgery started to be really difficult with me… Well, when she found out I’d gone to [GIC] she was really upset. (Richard, 63)

Examples of cisgenderism included participants being misgendered by their GP and reception staff, for example being referred to as ‘he’ rather than ‘she’ in public reception areas. Other participants observed a distinct change in their GP’s attitude after ‘coming out’ as trans, such as being told to wait outside, while notes were read or presenting as cold in manner. However, these encounters were the exception rather than the norm. In terms of organizational practice and record‐keeping, some individuals noted examples of misgendering in medical documents and correspondence, for example receiving correspondence addressed to them by the name assigned to them at birth (a form of ‘deadnaming’).

### 4.3 Uncertainty and delay in the health‐care system

Collective concern was expressed about the Welsh system for referral to a GIC in England (current at the time of interviews) as an ‘extra step to take in comparison to England’. When reflecting on their journeys to access gender‐affirming treatments, participants described themselves in a liminal space—always waiting in anticipation for the next point of contact and continually uncertain about their future. Progression through the health‐care system was perceived as a constant struggle with bureaucracy, compounded by low levels of knowledge about trans‐related health care amongst local professionals, including GPs and mental health practitioners.

#### 4.3.1 Delays and difficulties at the local level

There were variations in how quickly participants progressed through assessments and referrals via their local health board, indicating inconsistent procedures and timelines across boards. Some participants viewed themselves as ‘lucky’ as progression had been relatively smooth, while others described it as a ‘test’ they had to pass in order to be referred to a GIC or as a constant ‘fight for everything’ with clinicians. One repeated frustration was the reliance on local mental health gatekeepers to be able to progress to the GIC and the lengthy delays that ensued, varying between six and 18 months. Rebecca (53) experienced significant hurdles in convincing a mental health clinician she was a) transgender and b) not suicidal:

And I know what the criteria is to be accepted for surgery and everything, but he [practitioner] did turn around to say to me, ‘Well, you don’t look like you’re suicidal or anything like that’ and I said, ‘You’re an educated guy, I’m an educated person, why do I need to be suicidal to want to transition?’

Rebecca's account illustrates the power struggle present between the patient and practitioner where Rebecca is struggling to fit in the correct clinical boxes to be able to proceed with a GIC referral (according to the practitioner's current understanding). When appointments were cancelled repeatedly or referrals did not progress, some participants had ‘to make a fuss’ to ensure these problems were resolved. It was a common view that it was up to the individual to keep pushing against the system in order to receive referrals and access treatments and to know how to ask the ‘right questions’ to be able to move forward. In many instances, this meant relying on the knowledge of trans peers. Peer knowledge was invaluable whether it be through contact with other trans individuals in the local community or through information accessed via Internet‐based communities.

#### 4.3.2 Challenges and pressures when accessing GICs

Once referred to the GIC, participants described long waiting periods for appointments and frequent cancellations, further delaying treatment progression. For participants residing in Wales, having to deal with only one GIC results in a backlog, which led to increased waiting times to gain access. Frustrations with the system backlog led to a small number of participants with the financial means to consider private treatment. However, not everyone could afford this, as voiced by two participants who lived on low incomes generated from part‐time employment and social security.

The current system of accessing the GIC over the Welsh‐English border required lengthy travel and was deemed highly expensive for participants and for significant others providing emotional support. Many of the negative experiences recounted by participants revolved around long waiting times for appointments and frequent cancellations, sometimes on the day of the appointment (after travelling to the GIC) or the week before. The ‘impersonal’ approach of long waiting times coupled with repeated appointment cancellations extended to further waits for GIC staff to communicate with participants’ GP back home. Two people described waiting for over three months for letters to their GPs prescribing treatments.

Several participants reflected on the encouragement from GIC staff to change formally their names as part of a ‘real life test’ in order to progress to treatments. Some experienced this requirement as a positive step in their journey. Others were questioned by GIC staff about their choices, such as their chosen name:

I had changed my name … I have always been known as [chosen name]. So, when I did the deed poll, I changed my first name to […] and, at [GIC], I got massive grief … that it wasn’t a feminine enough name. (Diane, 67)

The power relationship between a GIC and its patients can result in people feeling that they have to be cautious about how they present, how they convey expected gender norms in actions and dress, and what they say. Two participants discussed the need to be hypervigilant when sharing personal information in case this worked against them, such as being identified as not meeting clinical criteria, that is not being ‘trans enough’.

A small group spoke positively about the GIC they had accessed, describing their interactions with staff as an ‘enjoyable experience’, a relatively quick progression of referrals and treatment, and ‘superb’ support. One person pointed to a ‘broken system’ that created problems such as long waiting lists, rather than a reflection of the performance of the clinicians within the service. When reflecting on what changes should be made to current provision, many participants envisioned a local cluster of integrated support through primary care services and believed that citizens in Wales should be cared for directly by GPs in local primary care services who have a more informed understanding of trans‐related health care.

## 5 DISCUSSION

In 2016, the UK Women and Equalities Committee identified significant systemic problems in delivering good standards of care to trans individuals accessing trans‐related health care, including knowledge levels and experiences of discriminatory treatment. The Committee pointed to GPs as one group lacking understanding of key areas including referral pathways to GICs and the prescribing of hormone treatment. In many ways, our findings echo these concerns. Participants’ reflections on pathways to accessing gender‐affirming treatments over the last ten years convey fractious and sometimes hostile relationships with GPs and other health‐care professionals, limited trans‐specific knowledge from clinical gatekeepers and repeated delays and hurdles in moving forward in their journey of transitioning. Alongside these concerns, there are some supportive interactions where participants receive gender‐affirming and highly validating responses in a timely manner.

Our findings bring acute attention to the psychological and emotional stressors trans adults in mid‐ to later life‐experience when navigating health‐care services, including experiences of overt transphobia and discriminatory treatment. This encompasses clinical pressures to conform to gender stereotypes, despite movement away from ‘real life tests’ by GIC services to more person‐centred approaches to supporting trans patients to transition socially on their own terms.^4^ For adults transitioning in their 50s and 60s, there is an underlying, chronological imperative of ‘running out of time’ to complete this life‐changing journey. Pearce discusses how trans lives within health‐care systems are marked by uncertainty and anticipation; there are elements of this present in our findings as participants recounted the cloud of uncertainty that enveloped their journey of seeking to transition medically.^16^ These findings chime with survey data reported by others in the UK^12^, ^17^ and the concerns raised by older adults in other European nations.^15^ Trans adults in the current study occupy the role of reluctant educators of GPs while relying on information gleaned from the Internet or through social connections with other trans individuals and groups.

Other authors have brought attention to the different ‘practices of care’ exercised across trans groups and networks^20^ and the role of community organizing for trans individuals living in a cisnormative social environment.^31^ Valderas argues that ‘recognising the importance of communities’ (p. 10) and the social groupings patients belong to is an integral strategy to enhancing patient engagement in the delivery of primary health care.^32^ A key recommendation from our study is the need for increased resourcing and support of local trans groups and networks by local, devolved and central governments. These groups often operate voluntarily without funding and are precariously placed in terms of providing on‐going support for individuals with limited social networks.

While the findings indicate a range of challenging and cisnormative interactions with professionals, the determination of individual participants to question, inform and assert their recognition and rights needs to be acknowledged. This sometimes required questioning medical expertise and was not in keeping with the more passive connotations of the patient role. The strength and resilience of older trans adults to question and challenge professionals and assert their care needs may wane in later years if their general well‐being is compromised by age‐related declines in physical and mental health. Literature on the intersection between ageing, gender identity and dementia reinforces this.^33^, ^34^ Another implication is the need for independent advocacy for trans individuals in later life if their care and support needs increase. Being a self‐advocate is a mentally and emotionally exhausting role, particularly after a lifetime accumulation of encounters with cisgendered assumptions and transphobic views and behaviours.

Within the third theme, there is promise that the current system will improve at a local level in terms of specialist health‐care services for trans citizens in Wales. There is a different kind of anticipation in participants’ accounts for a much‐improved service for future generations of trans individuals accessing these services. This aligns with current changes to NHS Wales services, following the recently launched Welsh Gender Team where individual patients will be able to access some specialist services.^30^ Alongside this, the Royal College of General Practitioners has called for a whole‐system approach to improving services for UK trans patients^35^. The current findings testify to the significance of enhancing local providers’ knowledge of GIC pathways and gender‐affirming treatments, inclusive of GPs, and to provide timely services in recognition of the years of anticipation older trans individuals have experienced over decades. We would add to this the importance of introducing compulsory learning sessions on gender identity and diversity across the life course within pre‐ and post‐qualifying training and education for health‐care professionals, of which some e‐learning materials currently exist within generic ‘equality and diversity’ modules online.

### 5.1 Limitations to the study

Our findings are generated from a small non‐representative sample, and participants were self‐selecting. This may have attracted a higher proportion of participants with negative experiences. Missing from the sample are the voices of trans individuals from black and minority ethnic (BAME) backgrounds; the findings do not account for inequalities compounded by the social intersections between old age, ethnicity and gender identity. For example, findings from the recent UK Government survey of LGBT people show older trans respondents (65 + years) were more likely to have experienced or been offered conversion therapy (20%) than younger respondents, with a higher number of BAME respondents (28%) reporting this.^9^ In this paper, we focused on those seeking to transition socially and medically and we cannot speak to the experiences of older individuals who are gender non‐conforming (eg identify as non‐binary). Separate research is warranted into their experiences of health‐care services that are heavily gendered. Likewise, more research is needed into trans experiences of high‐quality integrated care and the key differences between those who self‐fund and those who rely on public services.

## 6 CONCLUSION

Messages from this study speak to the importance of improving health‐care professionals’ knowledge of gender identity diversity across the life course and making changes at a systemic level in redressing cisnormative systems and practices. At a community level, trans consumers and groups could be supported to be actively involved in monitoring and evaluating the practices of local GP services in parallel with the whole‐system approach flagged above by the Royal College. Service audits by LGB&T consumers have been co‐produced and applied in social care settings^36^; there is scope to expand this model to the community auditing of primary care services by older trans individuals who bring expertise by experience from having accessed trans‐related health care. At a systemic level, the adoption of a gender affirmative model of health‐care delivery,^10^ coupled with an age‐friendly approach, is critical to building more accessible, responsive and supportive services that are co‐produced with trans groups and networks and include integrated support with social care and third sector support services. These initiatives require endorsement and resourcing from NHS bodies and their respective governments to ensure commitment from GPs and other health‐care professionals. Finally, more attention is needed on the perspectives of GPs and other clinicians on this topic to glean a deeper understanding of the individual, contextual and organizational factors that hinder practitioners from providing more gender‐affirming health care.

## CONFLICT OF INTEREST

All authors have no conflicts of interest to declare.

## Data Availability

Research data are not shared.

## Footnotes

^1^ For the purpose of this paper, we follow the definition of trans provided by Burns^37^ in her historical account of ‘Trans Britain’: an adjective ‘to describe anyone whose gender identity and/or presentation does not align stereotypically with the gender assigned them at birth’. (p. xvii) ‘Trans’ is a broad umbrella term ‘which encompasses all more specific words for gender non‐conformity including (but not limited to) transsexual people, non‐binary or genderqueer people and those who cross‐dress’ (p. xvii).
